# Introducing critical common dysregulated proteins in esophageal, gastric, and intestinal cancers 

**Published:** 2022

**Authors:** Babak Arjmand, Mohammadreza Razzaghi, Mostafa Rezaei Tavirani, Mohammad Rostami-Nejad, Majid Rezaei Tavirani, Reza Vafaee

**Affiliations:** 1 *Cell Therapy and Regenerative Medicine Research Center, Endocrinology and Metabolism Molecular-Cellular Sciences Institute, Tehran University of Medical Sciences, Tehran, Iran*; 2 *Laser Application in Medical Sciences Research Center, Shahid Beheshti University of Medical Sciences, Tehran, Iran*; 3 *Proteomics Research Center, Faculty of Paramedical Sciences, Shahid Beheshti University of Medical Sciences, Tehran, Iran*; 4 *Research Institute for Gastroenterology and Liver Diseases, Gastroenterology and Liver Diseases Research Center, Shahid Beheshti University of Medical Sciences, Tehran, Iran*; 5 *Faculty of Medicine, Iran University of Medical Sciences, Tehran, Iran*; 6 *Critical Care Quality Improvement Research Center, Faculty of Paramedical Sciences, Shahid Beheshti University of Medical Sciences, Tehran, Iran*

**Keywords:** Esophageal cancer, Gastric cancer, Intestinal cancer, Network analysis, AKT1

## Abstract

**Aim:**

The current study aimed to determine the common dysregulated proteins between esophageal, gastric, and intestinal cancers.

**Background:**

Though there are several documents about the role of AKT1 in promoting of esophageal, gastric, and intestinal cancers, there is not enough evidence about the dominant role of AKT1 relative to the other oncogene genes in the promotion of the three studied cancer types.

**Methods:**

One hundred proteins related to each of esophageal, gastric, or intestinal cancer were retrieved from the STRING database and interacted by Cytoscape software v 3.2.7. 2 to create the correlated interactomes. The network was analyzed by the “NetworkAnalyzer” application of Cytoscape to find the centrality parameters of the nodes. Results of network analysis and action map assessment were used to determine the common critical proteins between the three studied cancers.

**Results:**

One hundred proteins were extracted for each of the studied cancers. Among 42 common dysregulated proteins, 36 individuals were selected through network analysis and were screened through action map assessment. Eighteen proteins were introduced as the important common proteins. Finally, AKT1 was a candidate for the crucial dysregulated proteins common in the three analyzed diseases.

**Conclusion:**

The findings indicate that AKT1, relative to the other oncogene genes, is a suitable candidate to be evaluated in patients as a prediagnostic tool to reduce endoscopy and colonoscopy rates.

## Introduction

 Esophageal, gastric, and intestinal cancers are three important cancer types that are detected through aggressive methods such as endoscopy and colonoscopy. It is a great effort to establish a molecular-based method to diagnosis these types of cancers with economic and safe tools. Many diseases, such as diabetes and prostate cancer, are detectible by related biomarkers such as insulin and PSA, respectively (1-4). 

Network analysis is a useful method applied to screen large numbers of dysregulated proteins to detect the critical ones. The dysregulated proteins which are related to a disease connect together to form an interactome. Since each protein links to certain adjacent proteins based on its properties, network analysis distinguishes a few from the large number of queried proteins as the central ones. This is known as a scale-free network (5, 6). Several centrality parameters are applied to introduce the central nodes. One important central parameter is degree, which refers to the number of first neighbors of the node. The node with the highest degree value is called a hub node. Hub nodes are known as potent central nodes in network analysis (7). Betweenness is another important centrality parameter which is a function of the number of shortest paths in the formation of which the node participates. The node with a higher value of betweenness is known as a bottleneck node and is another powerful central node (8). Other centrality parameters, such as closeness centrality, are also used to identified the central nodes (9). 

Another useful method for assessing the queried proteins is action map analysis, in which the regulatory relationship between the studied proteins is explored. Different actions, i.e. activation, inhibition, and expression, are available for the evaluation of the studied proteins (10). Because the early detection of gastrointestinal cancers is important, identifying a common biomarker for these diseases was a main goal of the present research. Herein, the common dysregulated proteins between esophageal, gastric, and intestinal cancers are determined and investigated through network analysis and action map examination so as to find the critical ones. 

## Methods

The experimental approach is illustrated below:

**Figure:**
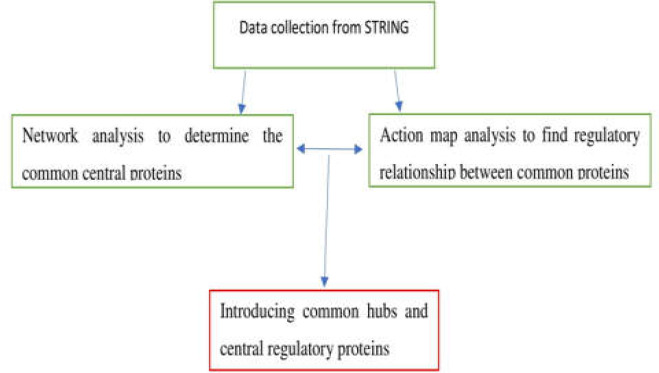


**Data Collection: **A total of 100 related proteins for esophageal, gastric, and intestinal cancers were downloaded from a “disease query” of the STRING database (11). 

**Protein-Protein Interaction Network Analysis: **The related proteins for each disease were included in a scale-free network in Cytoscape software to create an interactome (12). All interactomes were the analyzed using the “NetworkAnalyzer” application of Cytoscape software to assess the network topological properties. Nodes of the networks were placed in the layout based on degree values. Four centrality parameters, i.e. degree (K) (number of direct node connections), betweenness centrality (BC), closeness centrality (CC), and stress (number of shortest paths passing through a node), were determined for the nodes of the analyzed networks. CC and BC refer to the functions of the shortest paths which are related to the analyzed node. The top 10% of queried proteins based on degree value were considered as hubs for each network. Common proteins for the three cancers that included in the 10 top individuals based on degree value were identified as common hub nodes.

**Action Map Analysis:** To screen the queried proteins, common individuals between the three studied diseases, which were included in the 50% of top nodes (based on degree value) of the analyzed networks, were determined. These proteins were assessed through action map analysis using the CluePedia plugin of Cytoscape software to introduce the critical common dysregulate proteins for the three cancers. Activation, inhibition, and expression activity was considered for analysis of regulatory relationships among the investigated proteins. 

The common hub nodes, which were included in the resulted proteins of action map analysis, were introduced as critical dysregulated proteins in the three cancers. 

**Figure 1:**
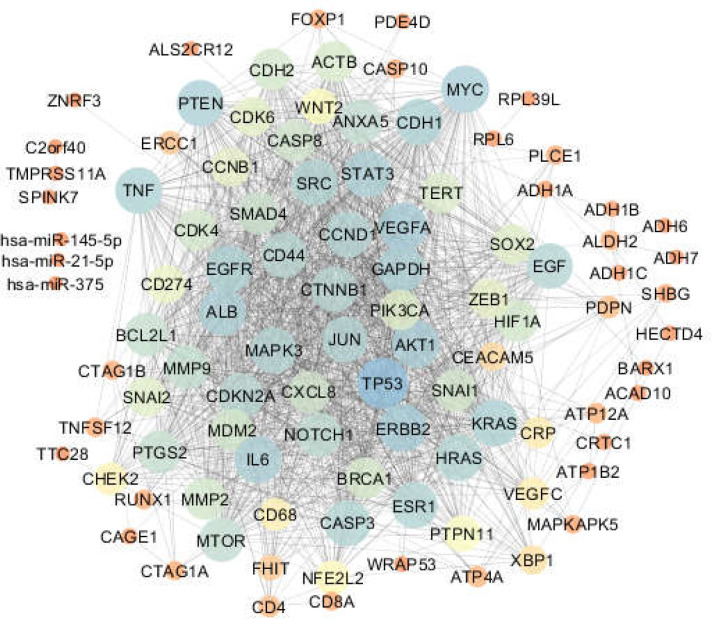
Esophageal cancer network (The nodes are laid out based on degree value). Colors from orange to blue refer to increment of degree value

**Figure 2:**
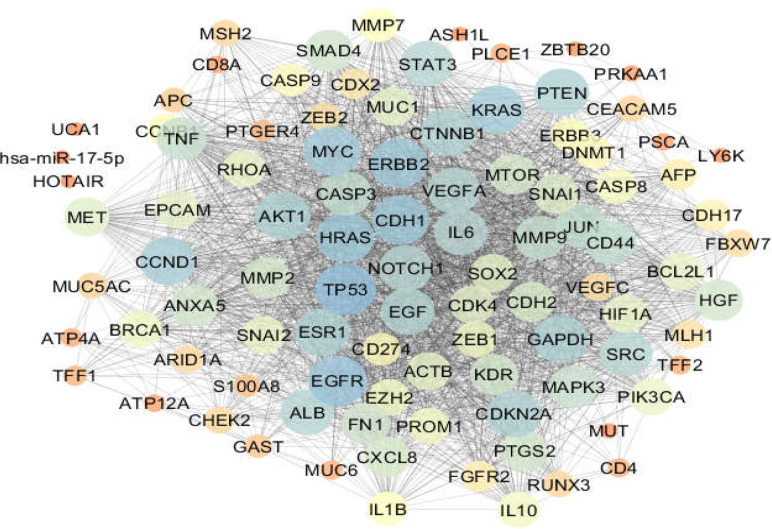
Gastric cancer network (The nodes are laid out based on degree value). Colors from orange to blue refer to increment of degree value

**Figure 3:**
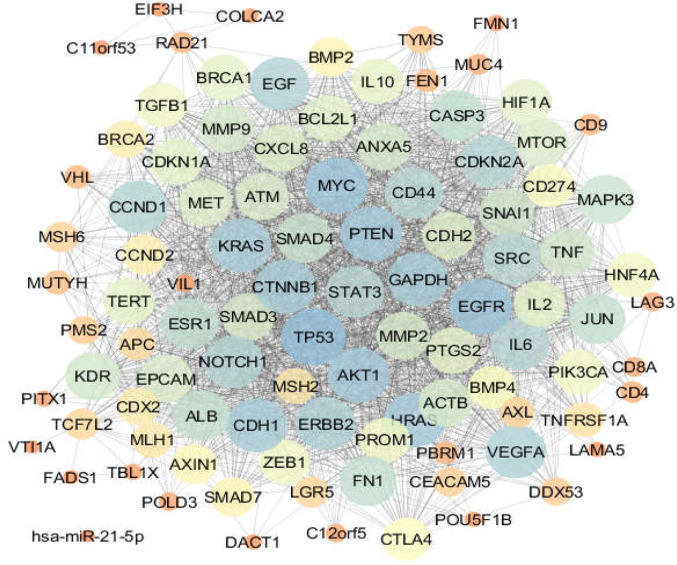
Intestinal cancer network, the nodes are in the layout based on degree value. Colors from orange to blue refer to increment of degree value

**Table 1:** Centrality parameters of AKT1 in the networks of esophageal, gastric, and intestinal cancers. Maximum values of degree for each analysis are showed in parentheses

	Degree	BC	CC	Stress
Esophageal cancer	59 (67)	0.009	0.684	1300
Gastric cancer	72 (72)	0.010	0.838	1776
Intestinal cancer	73 (79)	0.021	0.778	2992

## Results

One hundred proteins related to esophageal, gastric, and intestinal cancers were connected through 1484, 2164, and 2012 undirected edges, respectively, to form the related networks. The networks were analyzed, and the centrality parameters of the nodes were determined. The constructed networks were visualized based on degree values of nodes and are displayed in Figures 1-3. The current study identified 42 common proteins between the three studied cancers, of which 36 individuals were included in the top 50% of nodes (based on degree value) of the analyzed networks. Action map analysis revealed that ALB, MYC, 

 MAPK3, ACTB, VEGFA, and CASP3 were isolated in the expression map and were not considered for more assessment. Activation activity also indicated that MYC and ACTB are isolated from the other proteins. Inhibition activity showed that 17 proteins including ALB, MYC, ACTB, VEGFA, CASP3, and 12 other individual ones are not connected with the other proteins. Finally, 18 common critical proteins, i.e. AKT1, BCL2L1, CCND1, CDH1, CDKN2A, CTNNB1, EGF, EGFR, ERBB2, HRAS, IL6, JUN, KRAS, NOTCH1, SNAI1, STAT3, TNF, and TP53, remained as the core proteins of the three types of cancers. AKT1, PTEN, and GAPDH were identified as common proteins for the three cancers (common central nodes) and were included in the ten top individuals based on degree value. Only AKT1 was common between the results of action map analysis and common central nodes. The centrality parameters of AKT1 in the networks of the three studied cancers are tabulated in Table 1.

## Discussion

As the three studied cancers are quite similar (13, 14), the findings indicate that about 50% similarity is seen in the number of dysregulated proteins for each disease relative to the other type of cancers. Two aggressive methods, endoscopy and colonoscopy, are the usual detectors for diagnosing the three assessed cancers (15-17). Introducing new protein biomarkers can establish a useful method for detecting possible cancers in the esophagus, stomach, and intestine. 

Network analysis showed that 36 dysregulated proteins among the 42 most common ones are hub-like proteins (the number of hub proteins was more limited from 36 individuals). Action map analysis, which reveals the regulatory effects of nodes on the other proteins, indicated that 18 proteins among the 36 hub-like nodes interact with inhibition, activation, and expression activity. It seems that the key elements of a molecular detection method for esophageal, gastric, and intestinal cancers should be selected from among these 18 proteins. These screened proteins are compactly interacted in the action map, and it is impossible for each of them to be selected as a crucial individual. It seems another analysis is required to select a few of the 18 determined dysregulated proteins.

Centrality analysis showed that there are three common hub nodes, i.e. AKT1, PTEN, and GAPDH, for the three investigated cancers. The results further showed that PTEN and GAPDH are not included among the 18 proteins resulting from the screening experiments. The single common protein between the hubs and the 18 screened proteins is AKT1. Upregulation of AKT1 and cancer promotion have been reported by several researchers into liver gastric, colorectal, and breast cancers (18). 

AKT1 (v-akt murine thymoma viral oncogene homologue 1) kinase is a critical protein which plays an important role in proliferation and survival pathways in cancers (19). X. Zhang et al. reported that AKT1/mTOR pathway promotes gastric cancer (20). In their investigation entitled “HIF1α/miR-520a-3p/AKT1/mTOR feedback”, C. Pan et al. assessed two crucial processes (glycolysis and proliferation) of gastric cancer cells (21-23).

In their assessment, X. Liu et al. found that targeted therapy of the AKT kinase leads to inhibition of esophageal squamous cell carcinoma tumor growth (24). Our previous report indicated that a biomarker panel including AKT1, EGFR, and TP53 can be used to determine stages of esophageal cancer. The findings were achieved through network analysis (25). W. Wei et al. showed dysregulation of AKT1 in esophageal cancer which promotes the invasion of cancer (26). 

T. Liu et al. published a document about the role of the AKT pathway in promotion of cell proliferation in colon cancer (27). W. T. Wei et al. suggested that targeting MAPK1 and AKT1 leads to inhibition of cell proliferation in colorectal cancer (28). Based on the investigation of L. Jia et al., the miR-182/-135b/ST6GALNAC2/PI3K/AKT axis can be considered as a suitable prognostic and potential therapeutic biomarker in colorectal cancer (29). Based on the previous findings, AKT1 together with the other oncogene genes that are highlighted in gastrointestinal cancers, is a suitable biomarker for the mentioned cancers. 

In the present study, AKT1 was placed among the 100 proteins that were attributed to each type of esophageal, gastric, and intestinal cancers as a common critical dysregulated protein. It can be concluded that the current findings discriminate AKT1 among the other introduced oncogenes as a suitable biomarker for the three studied diseases.

AKT1, like the other oncogene genes, plays a role in some cancers: however, here it plays significant role in the three studied cancers relative to the other oncogene genes. The findings indicate that AKT1 is a biomarker candidate for esophageal, gastric, and intestinal cancers. If complementary and experimental investigations confirm this finding, checking AKT1 levels in patients can lead to a reduction in the performance of endoscopies and colonoscopies that are associated with improved diagnostic methods and economic advantages.

## Conflict of interests

The authors declare that they have no conflict of interest.
